# Metacognitive effects of attitudinal (in)congruence on social media: relating processing fluency, subjective knowledge, and political participation

**DOI:** 10.3389/fpsyg.2023.1146674

**Published:** 2023-07-17

**Authors:** Luna T. Frauhammer, German Neubaum

**Affiliations:** Department of Computer Science and Applied Cognitive Science, University of Duisburg-Essen, Duisburg, Germany

**Keywords:** attitudinal congruence, cross-cutting exposure, subjective knowledge, political participation, processing fluency, metacognition

## Abstract

**Introduction:**

Encountering political disagreements in our daily lives can discourage us from participating in democratic processes. To date, research has mainly focused on social motives or attitudinal mechanisms to explain this phenomenon. In the present study, we adopt a different approach and highlight metacognitive effects of attitudinal (in)congruence on processing fluency (i.e., perceived ease of processing) and subjective knowledge as well as their relationship with behavioral outcomes such as the intention to politically participate.

**Methods:**

In a pre-registered online experiment (*N* = 1,258), participants saw a political social media post with six opinionated user-generated comments. These comments either all matched (congruent condition) or contradicted (incongruent condition) participants’ personal opinions. Processing fluency, issue specific subjective knowledge, and intention to politically participate were then measured using established self-report scales.

**Results:**

In line with our hypotheses, the congruent stimuli evoked a higher feeling of processing fluency than the incongruent ones (*d* = 0.21). Furthermore, participants in the congruent condition indicated a higher intention to politically participate (*d* = 0.23) and rated their own knowledge on the topic as higher (*d* = 0.22) than participants in the incongruent condition—even though the factual knowledge gain should be equal in both conditions. Finally, we observed positive relationships between processing fluency and subjective issue knowledge (β = 0.11) as well as between subjective issue knowledge and issue-specific political participation (β = 0.43).

**Discussion:**

Our findings highlight the importance of considering metacognitive constructs such as subjective knowledge to explain political behaviors and suggest that attitudinal congruence influences the way we perceive our own knowledge and information processing.

## 1. Introduction

Humans tend to surround themselves with people who are similar to them (McPherson et al., 2001). Although this phenomenon was occurring long before the internet, the rise of social media platforms increased concern about the emergence of homogeneous opinion spaces (Terren and Borge-Bravo, 2021). Literature focusing on online homogeneity stresses possible negative outcomes such as fragmentation of the public sphere (Sunstein, 2017; Barberá, 2020) or political polarization (Kubin and von Sikorski, 2021). Yet, exposure to belief-reinforcing information might also have positive outcomes, such as increasing intended political participation (e.g., Wojcieszak et al., 2016), which is considered a core component of democracy (van Deth, 2016). Thus, to better assess possible consequences of such homogeneous sub-networks, it is important to further investigate the psychological effects of attitudinal congruence. Research has shown that people are less willing to participate in politics when they are surrounded by heterogeneous (vs. homogeneous) opinions (Mutz, 2002; Wojcieszak et al., 2016; Matthes et al., 2019). To date, this phenomenon has mainly been explained by social psychological processes such as effects of attitudinal congruence on attitude strength (Mutz, 2002; Visser and Mirabile, 2004) or social pressures (Mutz, 2002; Hayes et al., 2010). The present paper adopts a different approach and focuses on metacognitive effects of attitudinal congruence as well as their relationship with behavioral outcomes. More specifically, we expect attitudinal congruence to influence two important metacognitive constructs: the perception of processing ease or difficulty when reading new information (*processing fluency*; Oppenheimer, 2008) and a person’s self-perception of their own issue knowledge (*subjective knowledge*). This self-assessment represents a metacognitive component of knowledge and needs to be distinguished from the actual, objectively measurable knowledge a person holds (Carlson et al., 2009). Past research showed that subjective knowledge predicts behavioral intentions, e.g., the likelihood to politically participate (Wojcieszak et al., 2016; Lee et al., 2021a). The present paper reexamines this relationship from a metacognitive point of view. We therefore conducted a pre-registered experiment to test how varying levels of congruence between opinions displayed on social media and the viewer’s opinion affect processing fluency, subjective knowledge, and behavioral intentions.

## 2. Literature review

### 2.1. Effects of attitudinal congruence on political participation

The idea that our social environments influence the political attitudes and behaviors we hold is neither new nor specific to the social media context. As early as in 1944, sociologist Paul Felix Lazarsfeld and his colleagues described the role of social cross-pressures on individuals’ political behaviors (Lazarsfeld, 1968). He argued that more heterogeneous social networks were, for example, related to a later decision to vote. This research was followed by an abundance of studies investigating the effects of cross-cutting exposure on individual political attitudes and behaviors (Mutz, 2002; Scheufele et al., 2004; Quintelier et al., 2012; Feezell, 2016; Matthes et al., 2018; see Matthes et al., 2019 for a meta-analysis). Cross-cutting exposure thereby denotes “the disagreement in viewpoints encountered by individuals in their communication environments” (Matthes et al., 2019). In this study, we refer to cross-cutting exposure as a manifestation of attitudinal incongruence. Attitudinal (in)congruence, in turn, indicates the (mis)match between different persons’ attitudes toward the same attitude object, regarding attitude valence. Political participation, moreover, is a broad concept covering all kinds of citizen behaviors aimed at influencing political events (van Deth, 2016) and includes, for example, voting in an election, attending a demonstration, or writing a political social media comment. Since the construct is highly heterogeneous, scholars have attempted to find sub-dimensions of political participation. One possibility is the division in online and offline, as well as high-cost and low-cost, political behaviors (Knoll et al., 2020). Especially the distinction between online and offline political participation has received a lot of attention in the literature (Bakker and De Vreese, 2011; Bode, 2016; Lu et al., 2016; Knoll et al., 2020). In this context, the term “slacktivism” emerged, referring to low-effort online political behaviors which could make citizens feel politically engaged without having a real impact (Vitak et al., 2011). Although the general effects of attitudinal congruence on political participation should hold for both online and offline behaviors, the specific case of encountering attitudinal (in)congruence on social media could mainly influence other online, but not more distant offline behaviors. Based on this reasoning, we chose to exploratory test whether the hypothesized effects hold for both online and offline political participation items.

Cross-cutting exposure was originally assumed to discourage political participation (Mutz, 2002), a notion that was supported by a lot of research (Mutz, 2002; Valenzuela et al., 2012; Liu et al., 2013; Feezell, 2016; Lu et al., 2016; Moehler and Conroy-Krutz, 2016; Shi, 2016). Possible explanations for this effect include decreased attitude strength (i.e., reading incongruent opinions might cast doubts on the correctness of one’s own stance on an issue and reduce the strength with which one holds their own opinion; Wojcieszak et al., 2016) and social accountability pressures (i.e., people surrounded by incongruend opinions might be afraid to act on their beliefs due to fear of negative social consequences; Mutz, 2002). However, another line of research has challenged this view of the link between cross-cutting exposure and participation. Instead, it assumes that the confrontation with opposing opinions could actually increase political participation by fostering political learning and increased polarization (Matthes et al., 2019). This reasoning is supported by studies that find a positive relationship between cross-cutting exposure and political participation (Scheufele et al., 2006; Quintelier et al., 2012; Bae, 2013; Kim and Chen, 2015). Compatible with these contrasting findings, a recent meta-analysis did not find an overall relationship—neither positive nor negative—between cross-cutting exposure and political participation (Matthes et al., 2019). The authors therefore argued that cross-cutting exposure might not directly influence political participation and that effects observed in the literature could in fact be mediated. If cross-cutting exposure yields both positive and negative mediated effects on political participation, they can cancel each other out, thus leading to no overall effect. Future research, therefore, should not only investigate the relationship between cross-cutting exposure and political participation but also investigate the underlying mechanisms and possible mediators.

A further issue of the previous literature is that most studies rely on correlational data. Although these studies provide important insights and achieve high external validity, relying solely on correlational evidence is problematic since causal inferences cannot be scrutinized. Furthermore, correlational studies usually measure cross-cutting exposure through participants’ self-assessment, which may not be fully accurate. In the aforementioned meta-analysis, only few studies were experiments that actually manipulated cross-cutting exposure (Lu and Myrick, 2016; Moehler and Conroy-Krutz, 2016; Shi, 2016; Wojcieszak et al., 2016). Two of these experiments found cross-cutting exposure to negatively influence political participation (Moehler and Conroy-Krutz, 2016; Shi, 2016). Another experiment found no effect of cross-cutting exposure but a positive effect of pro-attitudinal exposure on political participation (Wojcieszak et al., 2016). Only Lu and Myrick (2016) found the opposite effect, indicating a positive relationship between cross-cutting exposure and political participation. However, the control condition in this experiment did not include any political messages. Therefore, the results of cross-cutting exposure leading to more political participation could be confounded with the sole effect of seeing a political message. To summarize, experimental data that allow causal inferences about the relationship between cross-cutting exposure and political participation are scarce. The experiments that do exist, however, suggest a positive effect of pro-attitudinal exposure as well as a negative effect of cross-cutting exposure on political participation. In a social media context, Neubaum and Krämer (2017a) observed no effect for attitudinal congruence of user-generated social media comments on willingness to participate in a discussion. There was, however, a significant effect of perceived attitudinal congruence, indicating that at least the perception of congruence can be important for explaining participatory behaviors. This study focused on a very specific form of participation, namely, the willingness to engage in online discussion. In the present experiment, we aim to investigate the effects of exposure to attitudinal (in)congruent social media comments on different forms of intended participation, both online and offline. We hypothesize the following:

*Hypothesis 1 (H1):* Exposure to attitudinal congruent (vs. incongruent) social media comments increases the intention to politically participate.^1^

### 2.2. Metacognitive processes as an explanation for the congruence–participation link

It is important not only to investigate the direct effect of cross-cutting exposure on political participation but to also focus on its underlying mechanisms. The literature assuming a negative effect of cross-cutting exposure on political participation has mainly suggested attitudinal and social mediators. Studies show, among other things, significant relationships between cross-cutting exposure and attitude extremity (Wojcieszak et al., 2016), attitudinal ambivalence (Mutz, 2002; Visser and Mirabile, 2004), and attitude certainty (Cargnino and Neubaum, 2022; Gill, 2022), all of which are indicators of attitude strength. Attitude extremity refers to how strongly a person feels about a specific political issue, attitudinal ambivalence indicates the extent to which a person might hold both positive and negative feelings on an issue, and attitude certainty denotes how confident a person feels about (the correctness of) their attitudes (Visser et al., 2006). These attitude strength related constructs could all be possible explanations for the relationship between attitudinal congruence and political participation. Furthermore, and in line with the spiral of silence theory (Noelle-Neumann, 1974), people in attitudinally incongruent environments could restrain from voicing their opinion or performing other political behaviors due to fear of social conflicts (Mutz, 2002; Hayes et al., 2010; Kim, 2016; Neubaum and Krämer, 2017b; Matthes et al., 2018).

People do not engage in political participation for social reasons alone, but also due to their convictions, in order to learn something new, or to persuade others (Weeks et al., 2015; Gil de Zúñiga et al., 2016; Winter and Neubaum, 2016). In our study, we therefore focus on cognitive factors that could explain the effect of attitudinal congruence on political participation. This notion receives initial support from a study on the effects of pro- and counter-attitudinal news on political participation (Wojcieszak et al., 2016). In this study, participants exposed to attitudinally congruent news stories rated their own issue understanding as higher than participants exposed to balanced news. Perceived issue understanding, in turn, was predictive of the intention to politically participate. The authors referred to this as a “cognitive mediator” (Wojcieszak et al., 2016). In the present paper, we want to further investigate these cognitive effects of attitudinal (in)congruence. However, we adopt a more nuanced terminology and use the term *meta*cognitive instead, since the participants’ perception of knowledge and not their actual knowledge is assumed to be the key variable.

Corresponding research uses different terminologies to describe this metacognitive construct, including perceived knowledge (Schäfer, 2020; Granderath et al., 2021), confidence in knowledge (Lee and Matsuo, 2018), political information efficacy (Kaid et al., 2007), and subjective knowledge (Leonhard et al., 2020; Lee et al., 2021a, 2021b). In this paper, we will adopt the term *subjective knowledge*. This can be considered as a counterpart to objective knowledge, which refers to the actual amount of information on an issue stored in memory. Although subjective and objective knowledge are usually significantly correlated, this association is only of moderate effect size (Alba and Hutchinson, 2000; Carlson et al., 2009), suggesting that the two aspects denote distinct—yet overlapping—constructs. We assume that people evaluate their own knowledge based on how their personal opinions match the opinions they encounter in their environment. A high level of attitudinal congruence with one’s environment could lead to the inference that one’s take on a political question is well-informed and based on a comprehensive evaluation. This reasoning can theoretically be embedded into active inference theory (Friston et al., 2017). Following this theory, the human brain constantly derives predictions about sensory inputs and strives to reduce the resulting prediction error. When new information is encountered, the recipient thus updates their beliefs about the causes of this data. Simulation studies showed that active inference theory can also be used to explain confirmation bias or the formation of echo chambers (Albarracin et al., 2022). Since the exposure to attitudinally congruent information evokes a smaller prediction error than the exposure to incongruent information, the recipients should feel reinforced and place more confidence in their prior opinions. They might then attribute this reinforcement onto their own expertise on the issue, resulting in a heightened feeling of subjective knowledge. Based on the above reasoning, we present the following hypothesis:

*Hypothesis 2 (H2):* Exposure to attitudinally congruent (vs. incongruent) social media comments will increase participants’ subjective issue knowledge.

#### 2.2.1. Behavioral effects of subjective political knowledge

Citizens’ objective political knowledge has been intensely studied in relation to political participation. While some scholars argue that political knowledge fosters political engagement (Johann, 2012), other researchers did not find a significant relationship between these two constructs (Cho et al., 2009; Dimitrova et al., 2014), which led to increased interest in the importance of subjective political knowledge. Indeed, several studies have shown subjective political knowledge to be a stronger predictor of general political participation than factual knowledge (Lee and Matsuo, 2018; Yamamoto et al., 2018; Lee et al., 2021a), as well as being related to young citizens’ likelihood to vote (Kaid et al., 2007) and participants’ willingness to contribute to a discussion (Schäfer, 2020). This suggests that subjective political knowledge is an important factor to explain political behaviors. This notion can be further strengthened by findings from different domains showing the importance of subjective issue knowledge to predict behavioral outcomes regarding, e.g., financial decisions (Hadar et al., 2013; Allgood and Walstad, 2016), environmental behaviors (Ellen, 1994; Pieniak et al., 2010), or purchase intentions (Berger et al., 1994).

From a theoretical point of view, it does not seem surprising that subjective knowledge is a more important predictor of behavioral outcomes than factual knowledge since it denotes the aspect of knowledge of which one is aware. At least when actions or behavioral decisions are to be based on knowledge, the self-perception of this knowledge is used to access whether one is well enough informed to make a decision and act on it (Dunning, 2011). Similar to this reasoning, research showed that subjective knowledge moderates the attitude–behavior relationship in the way that attitudes accompanied by high levels of subjective knowledge are more predictive of behaviors than attitudes accompanied by low levels of subjective knowledge (Berger et al., 1994). A more recent study showed that subjective knowledge moderates the relationship between attitude ambivalence and behavioral outcomes, indicating that attitude ambivalence only affected participants’ attitude-related behaviors when subjective knowledge was high compared to low (Wallace et al., 2020). Summarizing this research and theoretical reasoning, subjective knowledge might be an important construct to explain attitude–behavior discrepancies. While attitude valence and beliefs are important for predicting *which* behavior is executed, subjective knowledge seems to be important for predicting *whether* an individual decides to act at all. Following this reasoning, we assume that subjective knowledge is an important predictor of behavioral outcomes and formulate the following hypothesis:

*Hypothesis 3 (H3):* There is a positive relationship between subjective issue knowledge and intention to politically participate.

### 2.3. Explaining the relationship between attitudinal congruence and subjective knowledge

Provided that encountering attitudinal congruence increases people’s subjective knowledge, the question arises as to why. We propose two mechanisms that could explain this phenomenon: processing fluency and perceived issue controversiality.

#### 2.3.1. Processing fluency

Processing fluency denotes “the subjective experience of ease or difficulty with which we are able to process information” (Oppenheimer, 2008). A lot of research exists showing that perceptual (e.g., font type or size) as well as conceptual (e.g., jargon use, language difficulty) characteristics of stimulus material can alter participants’ perceived processing fluency (Oppenheimer, 2008; Song and Schwarz, 2008; Price et al., 2016; Shulman and Sweitzer, 2018; Shulman et al., 2020). In the present experiment, we propose that the attitudinal congruence between new information and a person’s prior opinion can also affect the feeling of processing fluency. The idea that information congruent to one’s own beliefs is easier to process than diverging viewpoints receives some support from past literature (Ziemke, 1980; Sherman et al., 1998; Gawronski and Brannon, 2019). However, empirical evidence explicitly testing this assumption is scarce and is mainly found in the literature on stereotypes. Here, research showed that stereotype-consistent information could be processed more easily than stereotype-inconsistent information, possibly because it fits better into existing schematic frameworks and thus is more readily understood (Sherman et al., 1998). Other research found that preference and value-inconsistent information evoked more effortful processing than consistent information (Ditto and Scepansky, 1998; Van Berkum et al., 2009). This reasoning might be transferable to attitudinal congruence, such that attitudinally congruent information could evoke a higher feeling of processing fluency than attitudinally incongruent information. Moreover, similar predictions could again be derived from active inference theory (Friston et al., 2017). Applying this theory to attitudinal congruence, it could be argued that attitudinal congruent information is processed more easily than incongruent information since it better matches the agent’s prior predictions, thus reducing prediction error. Following this theoretical and empirical literature, we propose the following hypothesis:

*Hypothesis 4 (H4):* Exposure to attitudinally congruent (vs. incongruent) social media comments will increase participants’ perceived processing fluency of reading the comments.

We also believe that the perceived processing fluency of reading the comments is related to participants’ subjective knowledge. Many studies link processing fluency to different metacognitive experiences such as judgments of learning or feelings of knowing (Schwarz, 2015). These findings can be explained by the feelings-as-information theory (Schwarz, 2012). This theory assumes that humans use feelings as an internal information source. One type of feelings that can be used as information are metacognitive experiences that arise during the processing of a stimulus. According to Schwarz (2004), we use so-called naïve theories to make sense of these experiences by attributing them to our own cognitive processes. For example, when learning material is easy to process, one could attribute this experience of processing fluency to one’s own knowledge about the topic, thus evoking a feeling of knowing (Koriat and Levy-Sadot, 2001; Shulman et al., 2020). We argue that political social media comments that are easy to process might trigger feelings of familiarity with the topic, thereby leading to higher subjective issue knowledge by making use of the naïve theory described above. This train of thought is supported by some research findings. Ryffel and Wirth (2020) showed that higher processing fluency of a news broadcast (generated by manipulating audiovisual characteristics) increased the viewers’ perceived political knowledge. In science communication, Scharrer et al. (2017) found their participants to have higher confidence in their own judgment about scientific topics after reading lay articles than after reading articles tailored to experts. Crucially, this effect could not be explained by different evaluations of topic complexity. Rather, the authors argued that the lay articles were easier to process, which in turn provoked the more optimistic confidence judgments. Finally, Shulman et al. (2020) showed that jargon use in scientific articles was predictive of processing fluency and, indirectly, related to scientific engagement such that less jargon predicted higher engagement. Based on this literature, we assume that processing fluency of political information is an important characteristic for metacognitive evaluations of political knowledge and propose the following hypothesis:

*Hypothesis 5 (H5):* There is a positive relationship between processing fluency and subjective issue knowledge.

#### 2.3.2. Perceived issue controversiality

Moreover, we propose perceived issue controversiality as a second mechanism that could explain the relationship between attitudinal congruence and subjective knowledge. Through reading attitudinally congruent social media comments, recipients could feel like there was only their own point of view on the topic, leading them to perceive the issue as less controversial. Perceived controversiality, in turn, was shown to negatively influence decision confidence (Scharrer et al., 2013), a construct related to subjective knowledge. Therefore, a reduced perceived controversiality through attitudinally congruent information should lead to higher subjective knowledge. However, Wojcieszak et al. (2016) could not observe the reversed effect, i.e., attitudinal incongruence leading to more perceived controversiality and reduced subjective knowledge. Given the lack of clear results, we ask the following research questions:

*Research Question 1 (RQ1): To what extent does exposure to attitudinally congruent (*vs. *incongruent) social media comments reduce participants’ perceived issue controversiality?*

*Research Question 2 (RQ2): To what extent is issue controversiality related to subjective knowledge?*

**Figure 1:**
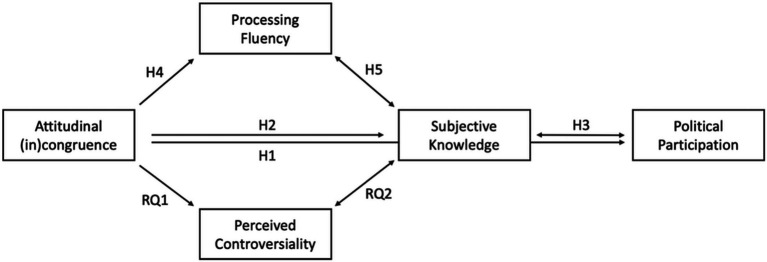
Overview of the overall theoretical model.

To summarize, the aim of the present experiment was to investigate metacognitive and behavioral effects of attitudinal congruence on social media. We therefore conducted an online experiment and presented participants with social media comments that either matched or contradicted their own opinions on controversial political issues. Figure 1 shows a graphical depiction of all hypotheses. Our results mainly support our hypotheses and underline the importance of considering metacognitive constructs when investigating attitudinal (in)congruence.

## 3. Materials and methods

### 3.1. Sample

In total, *N* = 1,258 participants completed the experiment (604 male, 647 female, five non-binary, two undisclosed). The sample size was based on an *a-priori* power simulation (see https://lunafrauhammer.github.io/Attitudinal_Congruence/Power for details). Participants were recruited through an online panel provider. The sample contained quotas for age and gender achieving a distribution roughly representative for German social media users. Participants’ ages ranged from 18 to 90 years, *M* = 40.89, *SD* = 14.37. Since the quality of data from panel providers was put under scrutiny (see, e.g., Webb and Tangney, 2022), we included two attention checks in the experiment (see “Procedure” section for more details). Participants who failed an attention check were excluded from completing the study. Furthermore, participants indicating that they never used social media were excluded from participation. Dropout rates were high: Of *N* = 2,641 participants who had started the experiment, *n* = 569 failed the first attention check, *n* = 451 failed the second attention check, and *n* = 85 participants indicated to never use social media. The other 278 missing participants left throughout the experiment. Since there was a systematic difference in dropout rates between the two conditions which might have corrupted the results, we reran our main analyses while controlling for the participants’ prior opinions and perceived issue relevance. The inclusion of these covariates did not change the results. Detailed information on these analyses as well as condition specific dropout rates and possible explanations for the systematic differences can be found in the Supplementary Material.

### 3.2. Design

The procedure was designed as a between-subject experiment with attitudinal congruence being the independent variable (exposure to social media comments that are congruent vs. incongruent with participants’ opinion). To increase generalizability, we further used two different political topics which, at the time of data collection (August 2022), both represented recent and controversial discussions in German politics: the implementation of a speed limit on German highways and the extension of the lifespan of German nuclear power plants. Participants were thus randomly assigned to one of four groups, with equal distribution being sought. This led to *n* = 316 participants in the speed limit-congruent condition, *n* = 314 participants in the speed limit-incongruent condition, *n* = 315 participants in the nuclear power-congruent condition, and *n* = 313 participants in the nuclear power-incongruent condition. Since we did not expect the topic to influence the results, both topics were jointly analyzed, leading to a one-factorial analysis design. For an overview of the experimental and analysis design please consult Supplementary Figure S1. The experiment was approved by the local institutional ethics review board. It was also preregistered on the open science framework.^2^

### 3.3. Procedure

**Figure 2:**
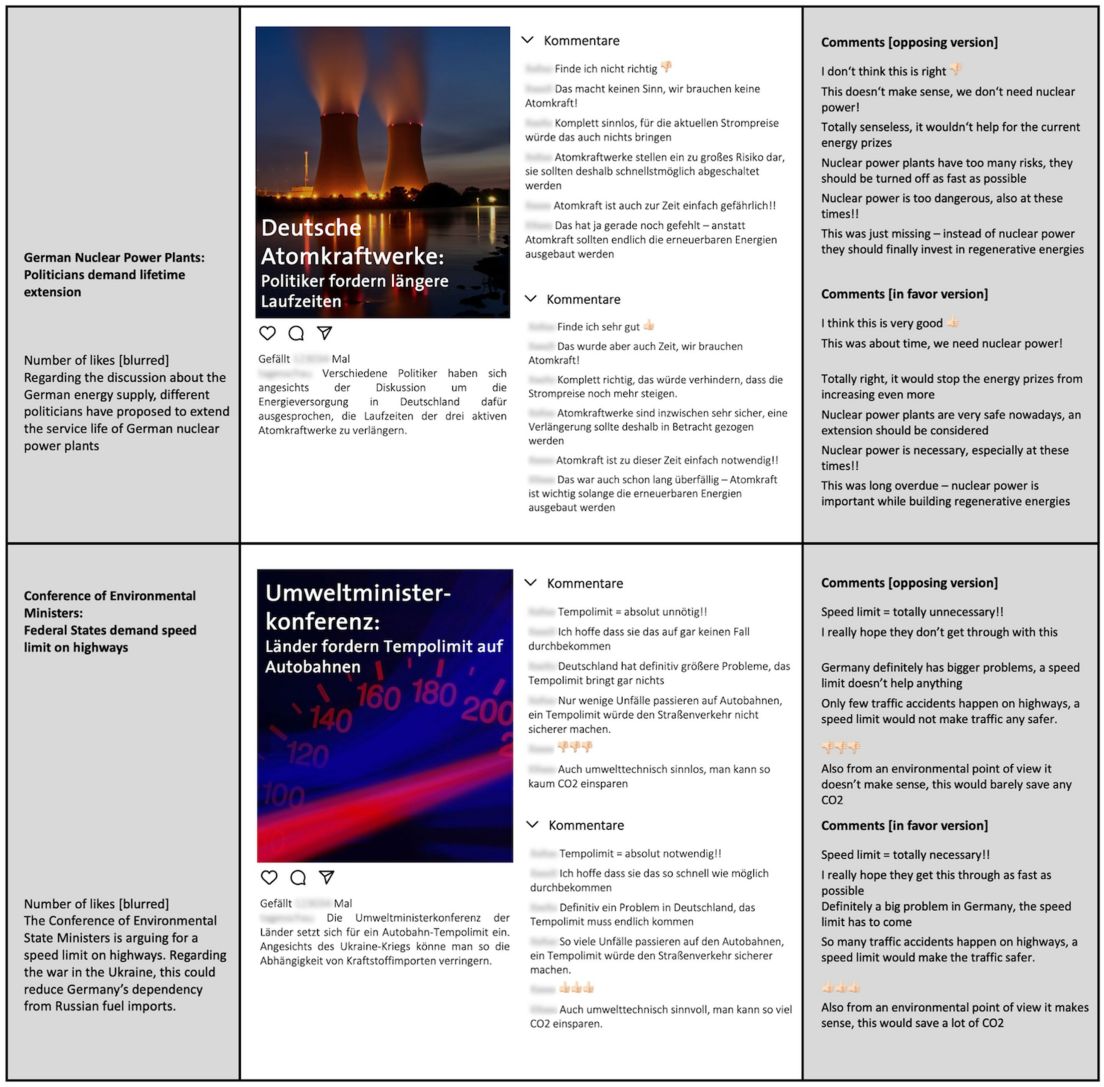
Original stimulus material with English translations in gray areas. Participants saw either opposing or supporting comments.

Participants attended the experiment on their personal computers by following a link. Participation *via* mobile devices (smartphones/tablets) was not possible. At the start of the experiment, participants provided written consent to participate in the experiment. Next, they were asked for their opinion about both topics on a scale from 1 to 7 (see section “Prior Opinions” for details). Participants who chose the middle category were further asked what their opinion would be if they had to decide. After a short block of bogus questions, participants were presented the social media post of their randomly assigned main topic with six corresponding user-generated comments (see Figure 2). Based on the experimental condition, these comments were either congruent or incongruent to the participants’ previously indicated opinion. Directly after the presentation of the post, participants answered the first attention check. This attention check was meant to ensure that participants had in fact read the stimulus material and asked whether the presented user-generated comments were in favor or against the discussed proposition. Participants who did not provide the correct answer were excluded from the remaining experiment. Following this attention check, participants answered the items measuring processing fluency of the comments and a number of bogus questions about the social media post. To ensure comparability of the two conditions, all participants then also saw the post about the other topic without user-generated comments. Following stimulus presentation, the remaining dependent variables as well as some control constructs were measured. The second attention check was embedded in the political participation items and asked participants to choose a specific answering option. All dependent variables except processing fluency of the comments were measured for both topics. The answers to the secondary topic questions (thus, regarding the social media post without comments) were meant to be used as the baseline condition. All exact items and English translations can be found on the Open Science Framework project page: https://osf.io/52kbs/.

### 3.4. Material

#### 3.4.1. Stimulus material

The stimulus material consisted of two fictional political social media posts with six corresponding user-generated comments. The two topics (speed limit on German highways and the extension of the lifespan of German nuclear power plants) were chosen to represent current controversies in German politics. The social media posts included a headline and a short first paragraph introducing the topic and a political proposition. These teasers were adopted from real newspaper articles with some changes (e.g., instead of names of politicians or political parties we chose neutral wordings). The user-generated comments were either all in favor or all against the proposition. Since we could only ensure equally distributed experimental conditions and not balanced distribution to the two stimulus sets (since this also depended on participants’ prior opinions), the comments were designed so that each comment had the same word count as well as similar wording and sentence structure in both sets. All comments were further pretested by 20 participants to ensure that their valence was perceived in the intended way. On a scale from 1 to 5, all chosen comments had mean valence ratings of either *M* > 4 for positive comments or *M* < 2 for negative comments. The mean ratings of all items were *M* = 4.62 for the positive and *M* = 1.40 for the negative comments. Figure 2 shows the original stimulus material with English translations.

#### 3.4.2. Prior opinions

**Figure 3:**
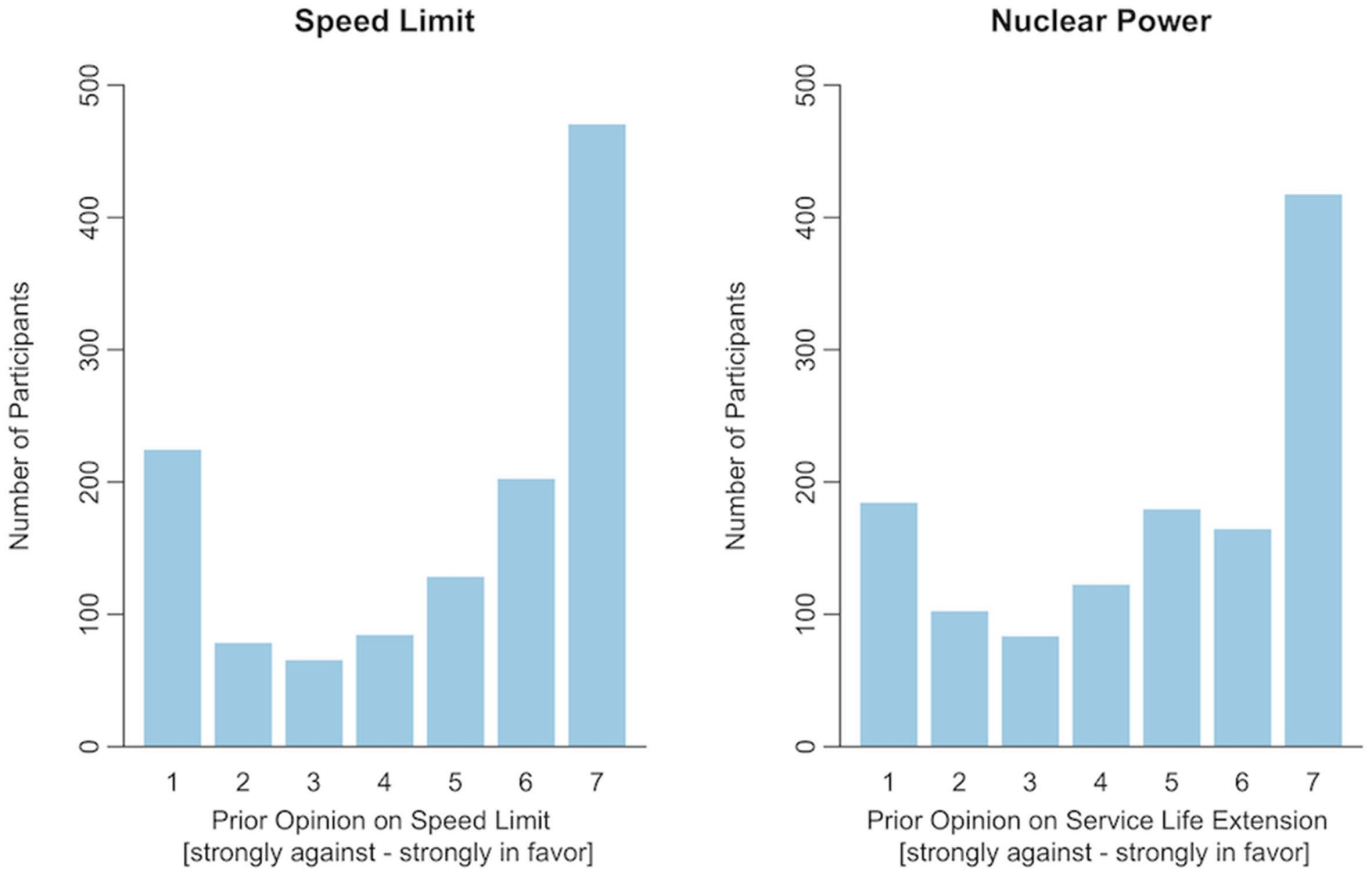
Distributions of prior opinions. Participants who chose answering option 4 (indicating no clear opinion) were further asked what their opinion would be if they had to decide (not included in this figure).

Participants’ prior opinions were assessed by asking them to indicate to what extent they agreed to the following statements: *A general speed limit should be imposed on German highways* and *The operating lives of the three active German nuclear power plants should be extended*. Participants who chose the middle answering option, thus indicating no opinion, were further asked what their opinion would be if they had to decide. For both topics, more participants indicated a positive than a negative opinion: Speed Limit: 850 (more) in favor, 408 (more) against; Nuclear Power: 823 (more) in favor, 435 (more) against. The complete distributions of participants’ prior opinions are shown in Figure 3.

#### 3.4.3. Dependent variables

All dependent variables were measured on 7-point scales. Supplementary Table S1 shows zero-order correlations of all dependent variables.

##### 3.4.3.1. Processing fluency of the comments

Perceived processing fluency of the comments was measured with five items (Graf et al., 2018). On a 7-point semantic differential scale, the items measured whether the comments were perceived as fluent vs. disfluent to read, effortful vs. effortless to process, difficult vs. easy to process, incomprehensible vs. comprehensible, and unclear vs. clear. The scale showed high internal consistency, *M*_SpeedLimit_ = 5.89, *SD*_SpeedLimit_ = 1.14, α_SpeedLimit_ = 0.91, *M*_NuclearPower_ = 6.05, *SD*_NuclearPower_ = 1.10, α_NuclearPower_ = 0.93.

##### 3.4.3.2. Perceived controversiality

Perceived issue controversiality was measured with six items (Scharrer et al., 2017). On a 7-point semantic differential scale, the items measured whether the issue was perceived as very one-sided vs. very many-sided, very reconcilable vs. very irreconcilable, not at all contradictory vs. very contradictory, not at all controversial vs. very controversial, not at all tense vs. very tense, and not at all conflicting vs. very conflicting. The scale showed acceptable internal consistency, *M*_SpeedLimit_ = 4.88, *SD*_SpeedLimit_ = 0.99, α_SpeedLimit_ = 0.78, *M*_NuclearPower_ = 5.08, *SD*_NuclearPower_ = 0.92, α_NuclearPower_ = 0.77.

##### 3.4.3.3. Subjective knowledge

Subjective knowledge was measured with six items (Schäfer, 2020). Participants were asked to indicate how much they agreed with each item on a 7-point scale. The items were: I know pretty much about the discussion on TOPIC; I am well informed about the discussion on TOPIC; I know a lot about the discussion on TOPIC; When it comes to the discussion on TOPIC, I know the relevant facts; I know the different standpoints on the discussion on TOPIC; I have a good overview of news on the discussion on TOPIC. The scale showed very high internal consistency, *M*_SpeedLimit_ = 4.73, *SD*_SpeedLimit_ = 1.45, α_SpeedLimit_ = 0.97, *M*_NuclearPower_ = 4.46, *SD*_NuclearPower_ = 1.54, α_NuclearPower_ = 0.97.

##### 3.4.3.4. Intention to politically participate

Issue-specific intentions to politically participate were measured with 11 items asking for both online and offline behaviors. The items were adopted from Kim and Ellison (2021) with some changes. On a 7-point scale, the items asked how likely the participants would perform one of 11 political behaviors in the next six months. The online political behaviors were: post a link to an article about TOPIC on social media; post a photo, video, or meme for or against TOPIC on social media; post their own opinion about TOPIC on social media; comment on someone else’s post on TOPIC; attend an online discussion event on TOPIC; and sign an online petition for or against TOPIC. The offline political behaviors were: attend an in-person discussion event on TOPIC; attend a demonstration for or against TOPIC; wear a sticker or sign for or against TOPIC; attend a political campaign for or against TOPIC; and contact a politician regarding TOPIC. The scale showed high internal consistency, *M*_SpeedLimit_ = 2.64, *SD*_SpeedLimit_ = 1.64, α_SpeedLimit_ = 0.95, *M*_NuclearPower_ = 2.57, *SD*_NuclearPower_ = 1.63, α_NuclearPower_ = 0.95.

One limitation of the above self-assessment scale is that it only includes hypothetical behaviors. Since survey responses about behavioral intentions can vary based on whether the items refer to very hypothetical versus more concrete, realistic scenarios (Scheufele et al., 2001), we chose to also include two more concrete items to measure behavioral intentions at the end of the experiment. First, we provided the participants with a link to further information on their main topic and tracked whether they clicked on it. However, since only very few participants followed the link (*n* = 22 in the speed limit group, *n* = 21 in the nuclear power group), we did not analyze these responses. Secondly, we asked participants whether they wished to be invited to a (fictional) online discussion event on their main topic, hosted by a public university. Responses to this question were more informative with *n* = 202 participants in the speed limit group and *n* = 190 participants in the nuclear power group wishing to be invited. We therefore performed additional analyses using this more concrete participation item below. Although this more behavioral item also captures the intention to participate (we do not know whether participants would, in fact, attend the event) and only looks at one specific behavior, we believe it to be an important addition to the self-assessment scale as it presents a more concrete and realistic scenario.

## 4. Results

Both topics were jointly analyzed for all hypotheses. Additional analyses for all hypotheses did not find any significant interaction effects with the main topic. We therefore computed new variables for every dependent variable that only contained the values of the main topic or secondary topic, respectively. To eliminate mean differences between the two topics, all variables were mean-centered within their respective topic group before being added to the joint variable. The reported values can thus be interpreted as absolute divergences from the respective topic mean. Mean centering did not notably change any of the results and we used this procedure to facilitate the joint interpretation of both issues. We further conducted an exploratory mediation analysis to evaluate the overall theoretical model we proposed. However, the reader should note that our experiment was not designed to test the causality of this model since it allows causal inferences only about hypotheses 1, 2, and 4 (group comparisons).

### 4.1. Hypothesis 1: effects of attitudinal congruence on intentions to politically participate

**Figure 4:**
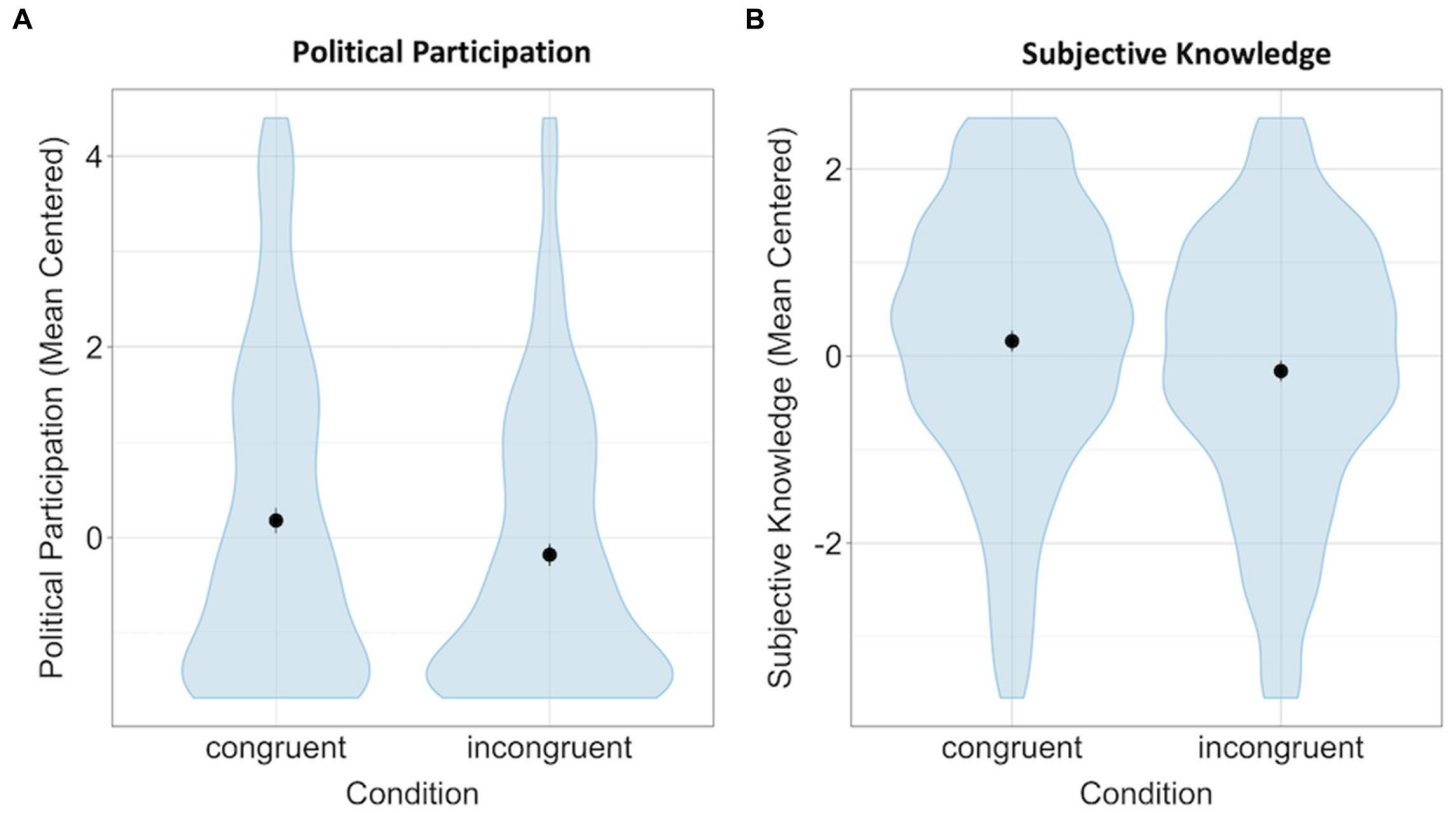
Violin plots for group differences in **(A)** political participation and **(B)** subjective knowledge. All values are mean-centered within their respective topic (original scale: 1–7). The black dots indicate the mean with its 95% confidence interval as error bars. The blue shaded area displays the kernel density plot of the data.

As hypothesized, self-reported willingness to politically participate was higher in the congruent than in the incongruent condition, *M*_congruent_ = 0.18 (*SD* = 1.7), *M*_incongruent_ = −0.18 (*SD* = 1.48; see Figure 4A). This numerical difference was significant, *t*(1234.9) = 4.00, *p* < 0.001, *d* = 0.23. The difference remained significant and increased in effect size when only including participants with a clear prior opinion, *t*(1043.2) = 4.25, *p* < 0.001, *d* = 0.26, *M*_congruent_ = 0.28, *M*_incongruent_ = −0.14. Online and offline political participation items were highly correlated, *r* = 0.84, and the group difference remained significant when only considering the offline political participation items, *t*(1229.8) = 3.79, *p* < 0.001, *d* = 0.21, *M*_congruent_ = 0.17, *M*_incongruent_ = −0.17. Since the variables were clearly not normally distributed, we further conducted a Wilcoxon-Rank-Test. This test also yielded a significant result, *W* = 219,680, *p* < 0.001. These results support H1.

**Figure 5:**
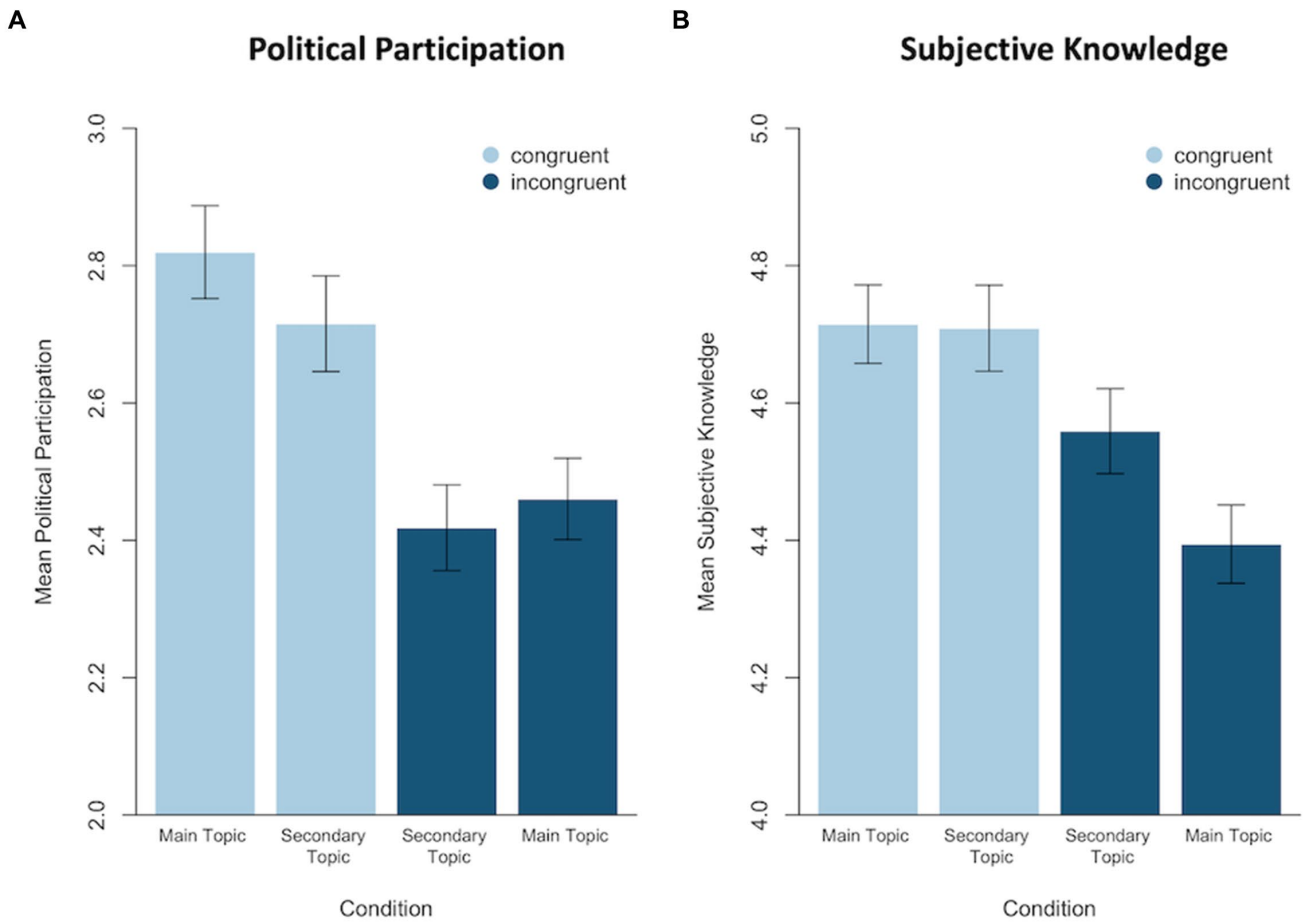
Mean differences between main topics and secondary topics regarding **(A)** political participation and **(B)** subjective knowledge. Main topic indicates that the participants saw a post with opinionated comments to this topic; secondary topic indicates that the participant saw a post without any comments to this topic (classification of congruent vs. incongruent is based solely on the main topic stimulus of each participant). Error bars indicate the standard error of the mean; scale: 1–7.

Following the significant overall effect, we next examined whether the group difference in political participation resulted from an increase in the congruent condition, a decrease in the incongruent condition, or both. For economic reasons and since this was not the main focus of our experiment, we did not include a separate baseline condition, but used each participant’s answers to the secondary topic (on which they saw the social media post without any comments and can be classified as neutral as opposed to congruent or incongruent) as baseline. We thus compared the dependent variables regarding the topic presented with comments (main topic) and without comments (secondary topic) *within* each condition, leading to a within-subject design (*cf.* Supplementary Figure S1). In a paired sample t-test, the main topic and secondary topic differed significantly in the congruent condition, *t*(630) = 3.18, *p* = 0.002, *d*_z_ = 0.13, meaning that participants in the congruent condition indicated a significantly higher willingness to politically participate for the topic on which they saw a social media post with congruent comments (main topic) than for the topic on which they saw a social media post without any comments (secondary topic). Which of the two topics (nuclear power vs. speed limit) was presented with or without comments was balanced between participants. This mean difference did not emerge in the incongruent condition, *t*(626) = 1.25, *p* = 0.213. Thus, the participants’ willingness to participate did not differ significantly between the topic on which they saw a social media post with incongruent comments (main topic) and a social media post without any comments (secondary topic). These results give preliminary evidence that the mean difference between the congruent and incongruent condition in political participation is due to an increase in participation in the congruent condition. However, these results need to be interpreted with caution: When looking at the mean values of each topic and condition, it becomes obvious that the participants’ political participation scores between the two conditions did not only differ in the main topic (for which the participants saw a social media post with comments, thus referring to our main analyses) but also in the secondary topic (for which the participants saw a social media post without any comments; see Figure 5A). This group difference in the secondary topic was statistically significant, *t*(1242) = 3.18, *p* < 0.001, *d* = 0.18. Implications of this unanticipated finding are outlined in the discussion section.

We finally tested for mean differences in the extra participation item, asking participants whether they wished to be invited to an online discussion event hosted by a public university. Slightly more participants indicated interest in the discussion event in the congruent vs. the incongruent condition, *n*_congruent_ = 210, *n*_incongruent_ = 182. However, a logistic regression with the experimental condition as a dichotomous predictor variable did not find this difference to be significant, log(*OR*) = −0.20, *OR* = 0.82, *z* = −1.63, *p* = 0.104.

### 4.2. Hypothesis 2: effects of attitudinal congruence on subjective knowledge

As hypothesized, subjective issue knowledge was higher in the congruent than in the incongruent condition, *M*_congruent_ = 0.16 (*SD* = 1.43), *M*_incongruent_ = −0.16 (*SD* = 1.42; see Figure 4B). This numerical difference was significant, *t*(1256) = 3.97, *p* < 0.001, *d* = 0.22. The difference remained significant and increased in effect size when only including participants with a clear prior opinion, *t*(1056.6) = 4.29, *p* < 0.001, *d* = 0.26, *M*_congruent_ = 0.33, *M*_incongruent_ = −0.03. These results support H2.

As in the previous section, we again conducted follow-up analyses comparing the topic presented with comments (main topic) with the topic presented without comments (secondary topic) within each condition to obtain a baseline measure. Regarding subjective knowledge, paired samples t-tests found no significant difference in the congruent condition, *t*(630) = 0.13, *p* = 0.897, meaning that these participants’ subjective knowledge did not differ significantly between the topic on which they saw a social media post with congruent comments (main topic) and the topic on which they saw a social media post without any comments (secondary topic). It was, however, significant in the incongruent condition, *t*(626) = −3.89, *p* < 0.001, *d*_z_ = 0.15, meaning that participants in the incongruent condition indicated a significantly lower subjective knowledge for the topic on which they saw a social media post with incongruent comments (main topic) than for the topic on which they saw a social media post without any comments (secondary topic). These results provide tentative evidence that the mean difference between the congruent and incongruent condition in subjective knowledge is due to a decrease in the incongruent condition. However, these results again need to be interpreted with caution, since we again found a significant, yet smaller, group difference in subjective knowledge regarding the secondary topic, *t*(1255.9) = 1.71, *p* = 0.043, *d* = 0.10 (see Figure 5B). Implications of this unanticipated finding are outlined in the discussion section.

### 4.3. Hypothesis 3: relationship between subjective knowledge and intentions to politically participate

**Figure 6:**
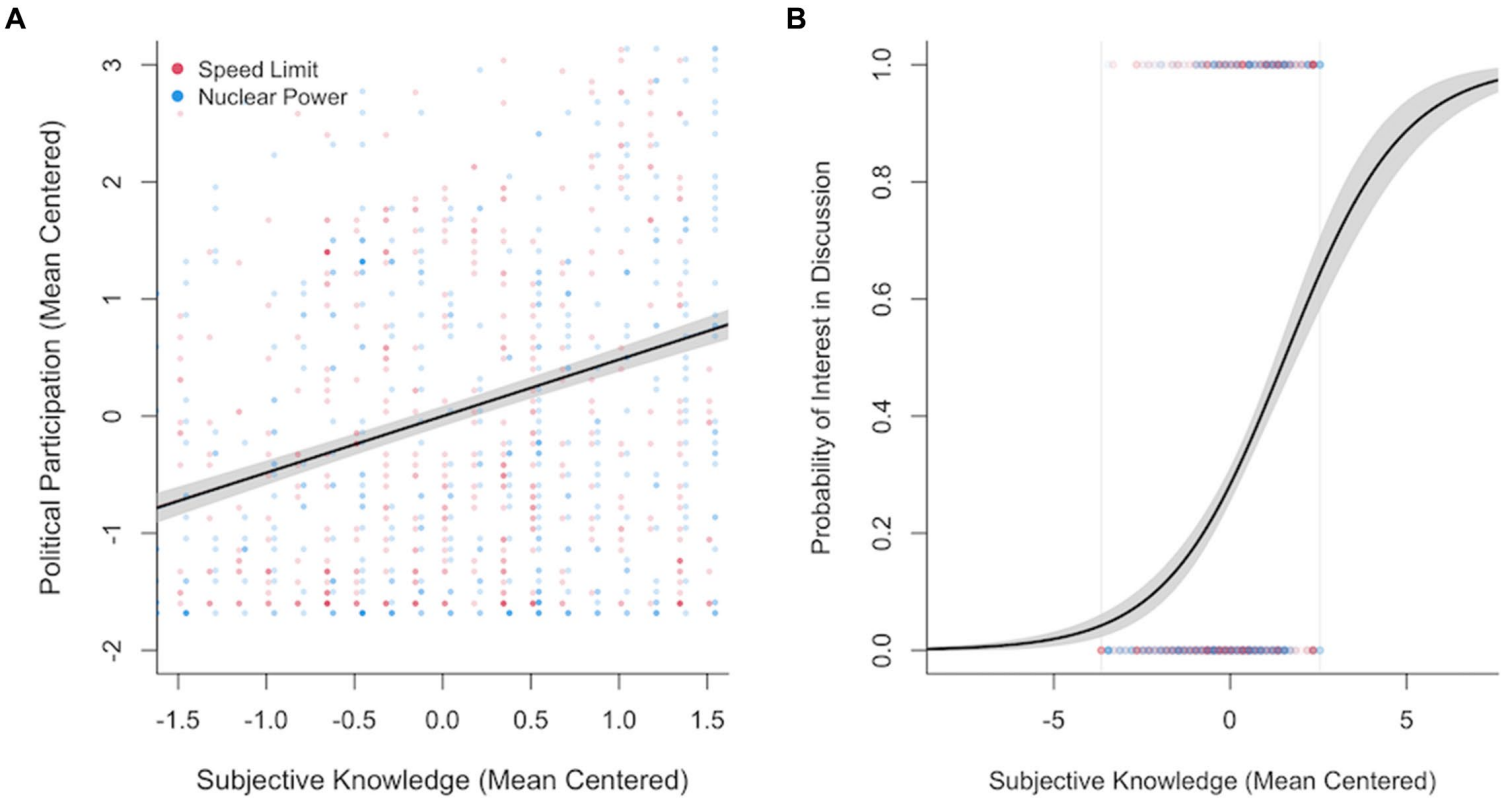
Results of regression analyses. **(A)** Linear regression of subjective knowledge on political participation. All values are mean-centered within their respective topic (original scale: 1–7). The shaded area indicates the 95% confidence interval. **(B)** Logistic regression of subjective knowledge (mean-centered) on whether or not the participants wish to be invited to an online discussion event. The regression line indicates the predicted probability of choosing yes (i.e., wishing to be invited). The vertical gray lines indicate the area of observed subjective knowledge values. The shaded area indicates the 95% confidence interval.

Hypothesis 3 assumed a positive relationship between subjective issue knowledge and issue-specific political participation. A simple linear regression established a positive, significant relationship between the two variables, *b* = 0.48, *SE* = 0.03, 𝛽 = 0.43, *t*(1256) = 17.02, *p* < 0.001. Figure 6A illustrates this relationship.

We also tested this relationship using the extra participation item measuring interest in a discussion event. A logistic regression established a positive, significant relationship between subjective issue knowledge and the probability of wanting to be invited to the discussion event, log(*OR*) = 0.60, *OR* = 1.82, *z* = 11.08, *p* < 0.001. Figure 6B illustrates this relationship.

### 4.4. Hypotheses 4 and 5: effects of attitudinal congruence on processing fluency

Participants rated the processing fluency of reading the user-generated social media comments as higher in the congruent opposed to the incongruent condition, *M*_congruent_ = 0.11 (*SD* = 1.05), *M*_incongruent_ = −0.11 (*SD* = 1.17). This numerical difference was significant, *t*(1241.4) = 3.64, *p* < 0.001, *d* = 0.21. These results support H4. H5 predicted a positive relationship between perceived processing fluency and subjective knowledge. This hypothesis is supported by the results of a linear regression analysis, *b* = 0.14, *SE* = 0.04, 𝛽 = 0.11, *t*(1256) = 3.84, *p* < 0.001.

### 4.5. Research questions 1 and 2: effects of attitudinal congruence on perceived controversiality

In RQ1, we were interested in whether there were any group differences in perceived controversiality of the topic. The topics were perceived as more controversial in the incongruent than in the congruent condition, *M*_congruent_ = −0.08 (*SD* = 0.97), *M*_incongruent_ = 0.08 (*SD* = 0.86), *t*(1238.2) = −3.00, *p* = 0.001, *d* = 0.17. Finally, we investigated whether perceived controversiality and subjective knowledge were related. We found a small, positive relationship between the two variables, b = 0.14, *SE* = 0.04, 𝛽 = 0.09, *t*(1256) = 3.17, *p* = 0.002.

### 4.6. Exploratory analyses: mediation analysis

**Figure 7:**
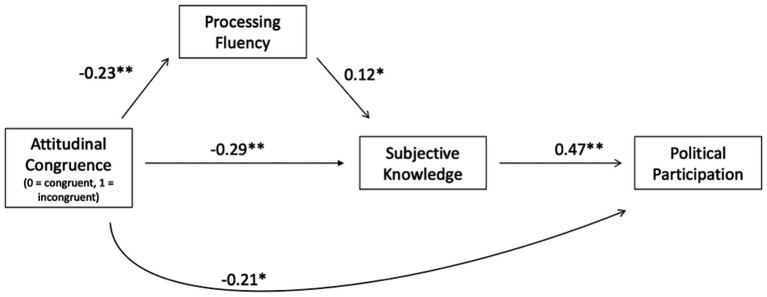
Results of mediation analysis. RMSEA = 0.10 [0.05; 0.15]; CFI = 0.96; SRMR = 0.03; ** denotes *p* < 0.001, * denotes *p* < 0.05.

In addition to our preregistered analyses, we further conducted an exploratory mediation analysis based on our overall theoretical assumptions. However, our research design does not allow causal conclusions regarding the relationships between subjective knowledge and political participation, and processing fluency and subjective knowledge, respectively. The reader should keep this in mind when consulting the below analyses. Figure 7 shows the results of the mediation analysis. The indirect effect of experimental condition on political participation through subjective knowledge was significant, *b* = −0.14, *z* = −3.54, *p* < 0.001. The indirect effect of experimental condition on subjective knowledge through processing fluency was also significant, *b* = −0.03, *z* = −2.44, *p* = 0.015.

## 5. Discussion

The aim of the present study was to investigate the metacognitive and behavioral effects of being exposed to attitudinal (in)congruent political information on social media. In accordance with our hypotheses, willingness for political participation and subjective issue knowledge were higher after being exposed to politically congruent social media comments than after receiving incongruent ones. Furthermore, subjective knowledge and political participation were positively related. Supporting the remaining hypotheses, we observed significant group differences in processing fluency and perceived controversiality as well as significant, yet small, relationships between these variables and subjective knowledge.

### 5.1. Effects of attitudinal congruence on political participation and subjective knowledge

In accordance with our first hypothesis, participants exposed to attitudinally congruent social media comments indicated a higher willingness for political participation than did participants exposed to attitudinally incongruent social media comments (*d* = 0.23). Furthermore, this group difference was evident not only for online but also for offline political participation, implying that the effects of information we receive on social media are not limited to online behaviors but can also affect how we act offline. Our results, showing that people receiving belief-congruent information are more willing to politically participate than people receiving incongruent information, are in line with other experimental research on the relationship between cross-cutting exposure and political participation (Moehler and Conroy-Krutz, 2016; Shi, 2016; Wojcieszak et al., 2016). When looking at the entire literature, however, this relationship becomes less clear since past studies found both negative and positive as well as no relationships between cross-cutting exposure and political participation (e.g., Mutz, 2002; Quintelier et al., 2012; Bae, 2013; Feezell, 2016; Matthes et al., 2019). One possible reason for these discrepancies could lie in different definitions and operationalizations of cross-cutting exposure (Klofstad et al., 2013) as well as different research methods. In our study, we defined attitudinal (in)congruence as the (mis)match between new information and participants’ prior opinions. By only including either congruent or incongruent comments (leading to homogeneous stimulus material in both conditions), we looked at extreme forms of attitudinal congruence. It is also possible to vary the heterogeneity of information by including different opinions in the stimulus material, which, however, was not the goal of the present research. We also chose an experimental approach and manipulated what participants saw instead of using self-reports. Such methodological choices could to some extent explain the inconsistent results on the attitudinal congruence–participation link. Another explanation for diverging results could lie in the operationalization of the dependent variable, that is, political participation. While we found a positive effect of attitudinal congruence on intentions to politically participate for the self-assessment scale, this group difference was not observable when asking participants for their interest to participate in an online discussion event. Research on the spiral of silence theory found that participants’ answers to self-assessment items on behavioral intentions vary based on how concrete and realistic the presented scenario is (Scheufele et al., 2001). These results indicate a need to include more concrete and realistic scenarios to measure political participation intentions in order to rule out that the effects found in the literature are limited to artificial survey responses.

From a societal perspective, our results depict an interesting field of tension regarding the effects of online attitudinal congruence. In our experiment, participants being exposed to social media comments that reinforced their prior opinions indicated a higher willingness for political participation than did participants exposed to incongruent comments. This suggests that attitudinal congruence—which normally is depicted as rather non-desirable in the literature—can also have positive outcomes, since political participation constitutes a core element of democratic societies (van Deth, 2016). However, if these higher levels of participation come at cost of communication between people holding different viewpoints, another key component of democracy is corrupted. After all, democracy is about exchanging and accepting different opinions, and searching for the best solution for everyone (Habermas, 1991; Dahlberg, 2005). If only those surrounded by likeminded others are willing to politically participate, this could lead to a silencing of minority opinions (Noelle-Neumann, 1974). It should thus be considered a priority to ensure that everyone—including those holding minority opinions—feels confident enough to raise their voices and participate in society. At the same time, enabling people to interact in spaces that are to some extent homogeneous could increase their mutual mobilization and group cohesion, also allowing them to pursue their common cause (O’Hara and Stevens, 2015).

Our present findings also provide a preliminary explanation for the effect of attitudinal (in)congruence on participation: Those who were exposed to attitudinally congruent social media comments rated their subjective knowledge as higher than those who saw incongruent comments (*d* = 0.22)—even though the factual knowledge gain should be very low and above all equal in both conditions. While these results are in line with prior findings, showing that exposure to attitudinally congruent news led to higher ratings of subjective knowledge than reading balanced news (Wojcieszak et al., 2016), they extend this research by showing that social media comments (which are mostly not crafted on the basis of journalistic ideals and which convey much less information) can also foster our sense of being more knowledgeable. The perception of an attitudinally congruent opinion climate can thus not only reinforce one’s prior opinions (Visser and Mirabile, 2004) but also resulted in a higher perception of one’s own knowledge than did the perception of incongruence. This could have the effect that people in homogeneous opinion spaces feel better informed than people in more heterogeneous environments—without actually possessing more knowledge. Such a miscalibration of subjective and objective knowledge might have worrisome effects such as, for example, discouraging learning and information searches on the issue or even favoring the preservation and distribution of false information (Williams Kirkpatrick, 2021). In fact, research has found positive relationships between knowledge miscalibration and the holding of anti-consensus views on scientific and political issues (Light et al., 2022), which might be explained by a reduced sensitivity to new evidence (Rollwage et al., 2018, 2020; Wood and Lynch, 2002).

From a more optimistic perspective, and following our theoretical assumptions, higher subjective political knowledge might also increase political participation by making people feel confident enough to act on their attitudes. Indeed, we observed a positive, medium-sized relationship (*r* = 0.43) between participants’ subjective issue knowledge and willingness for issue-specific political participation, supporting our third hypothesis. This relationship did not only arise for the hypothetical political participation scale but also when regarding whether participants wished to be invited to an online discussion event, representing a more concrete scenario. These results are in line with prior findings (Lee and Matsuo, 2018; Yamamoto et al., 2018; Lee et al., 2021a) and provide tentative support for the assumption that subjective knowledge might favor behavioral outcomes such as political participation. However, the present as well as past findings do not permit causal conclusions. It could therefore also be that more political participation leads to higher ratings of subjective knowledge or that a third variable, like interest, affects both constructs. To rule out these competing interpretations, further experimental evidence is needed on the subjective knowledge–political participation relationship.

#### 5.1.1. Does congruence increase or incongruence decrease political participation?

We intended to use the responses to the posts presented without any comments as a control condition for the group comparisons of subjective knowledge and political participation. This seemed adequate since participants saw only a social media post but no user-generated comments on these issues. When comparing the topic with comments (primary topic) and without comments (secondary topic) within each condition (resulting in a within-subject design), we found a significant effect for political participation in the congruent condition and a significant effect for subjective knowledge in the incongruent condition. This would imply that the group difference in political participation arose due to an enhancing effect of attitudinally congruent information, while the group difference in subjective knowledge arose from a decreasing effect of attitudinally incongruent information. Since these effects are opposite for both constructs, these findings question the mediating role of subjective knowledge that we proposed in our theoretical reasoning. However, the results of these within-subject comparisons must be interpreted with caution. As already described in the “Results” section, we found unanticipated group differences for the posts presented without comments. We explain these group differences as carry-over effects of the experimental manipulation, which are known hazards to within-subject designs. This would mean that the experimental manipulation of the primary topic also affected the responses to the secondary topic. This could be further amplified by the close temporal proximity of both issues and the limited stimulus set of only two posts. When interpreting the effects not as artifacts from this experimental situation, they appear even more unsettling since this would imply that perceiving attitudinally congruent information to one issue not only increases one’s subjective knowledge regarding this topic but also regarding related issues. This could further expand the aforementioned knowledge miscalibration resulting from attitudinally congruent opinion spaces. However, independent of the specific interpretation, these unanticipated results question the suitability of the secondary topics as a control condition.

### 5.2. Processing fluency and perceived controversiality

As another goal of our experiment, we investigated processing fluency (i.e., perceived ease of processing; Oppenheimer, 2008) and perceived issue controversiality as possible explanations for the effect of attitudinal congruence on subjective issue knowledge. In our study, attitudinally congruent social media comments evoked a higher feeling of processing fluency than did attitudinally incongruent ones (*d* = 0.21). This implies that attitudinal congruence might affect the very way we process information, or at least our self-perception of this process. Although some authors have discussed related phenomena such as easier processing of stereotype or belief-consistent information (Ditto and Scepansky, 1998; Sherman et al., 1998; Gawronski and Brannon, 2019), research on this issue is still very scarce. Therefore, further research is needed to replicate this effect and to test whether it only holds for the perceived ease of processing (i.e., processing fluency), or whether attitudinally incongruent content might in fact hamper information processing. We further observed a positive relationship between processing fluency and subjective knowledge (*r* = 0.10), supporting our fourth hypothesis. However, this effect was very small, with processing fluency explaining only 1% of variance in subjective knowledge. Although such small effects could still be of practical relevance if they accumulate due to repeated exposure or when considered at population level (Funder and Ozer, 2019), the observed relationship should not be over-interpreted. The lack of a stronger relationship could arise from our selection of topics. These both represented current discussions in German politics regarding which participants most likely already possessed prior knowledge and experiences on which they could base their subjective knowledge evaluations. It is therefore somewhat reassuring that a single experience of processing ease or difficulty did not fundamentally alter a person’s subjective knowledge. Lastly, this relationship can yet again only be interpreted correlationally, and one should consider different possible underlying mechanisms. For example, it could be that the relationship between processing fluency and subjective knowledge is spurious and can be explained through effects of objective knowledge on both variables, which we did not assess.

Finally, we tested in an exploratory manner for the effect of attitudinal (in)congruence on perceived controversiality, as well as its relationship with subjective knowledge. We again found significant group differences with higher perceived controversiality in the incongruent than the congruent condition (*d* = 0.17). This effect, however, was again very small and of questionable practical relevance. Perceived controversiality was further disqualified as a possible mediator since it correlated weakly positively (and not negatively) with subjective knowledge (*r* = 0.08). This positive relationship is somewhat surprising since it means that people who perceive an issue to be more controversial also believe they have more knowledge about it. One explanation for this could be that participants who felt they knew a lot about the issues were more likely to also know different viewpoints on it and thus perceived it as more controversial. In fact, one item in the subjective knowledge scale asked about knowledge on different standpoints on the topic. In order to indicate high subjective knowledge in this item, one needs to perceive the issue as somewhat controversial or else there would be no different standpoints. In total, however, the effects of perceived controversiality are even smaller than the effects of processing fluency and, in our opinion, of limited importance in the present experiment.

### 5.3. Theoretical implications

The present research offers some theoretical implications. First and foremost, it highlights the importance of considering metacognitive effects of attitudinal (in)congruence. In our experiment, exposure to congruent versus incongruent social media comments altered both the evaluation of one’s knowledge as well as the perception of ease with which this information was processed. Regarding the effect on subjective knowledge, this could lead to the emergence of a systematic knowledge miscalibration: People embedded in homogeneous networks might perceive themselves as more knowledgeable than people in more heterogeneous networks—without actually possessing more objective knowledge. Since subjective knowledge was also linked to behavioral intentions, our findings further provide a tentative explanation for the cross-cutting exposure–political participation link (Wojcieszak et al., 2016). Thus, only focusing on social or attitudinal mediators might not cover all the psychological mechanisms at play. Although the observed effect sizes of the group differences in subjective knowledge and political participation are appraised as small by traditional thresholds (*d* ≈ 0.20; Cohen, 1992), they seem substantial when considering that they are based on a single exposure to six social media comments. If people are constantly embedded in homogeneous opinion networks, these effects might become larger and more relevant (Funder and Ozer, 2019). Moreover, the association between subjective knowledge and political participation was of substantial effect size (*r* = 0.42), indicating that these two constructs are strongly connected. Further research should thus investigate the underlying mechanism of this relationship and consider cognitive motives for political participation more strongly.

Finally, our findings concerning processing fluency are very intriguing and suggest that attitudinal (in)congruence may not only influence higher-level reasoning processes regarding, e.g., attitude strength (Visser and Mirabile, 2004; Cargnino and Neubaum, 2022) or social consensus evaluations (Neubaum and Krämer, 2017b), but might alter the very process of information processing. Participants in our experiment indicated that the processing of congruent information felt easier than the processing of incongruent information. As such, our findings offer possible cognitive bases for effects such as selective exposure (Friedman and Sears, 1965) or confirmation bias (Klayman, 1995), since the feeling of processing ease is heuristically linked to judgments of truth and liking (Schwarz, 2012).

### 5.4. Limitations

Like every empirical study, the present experiment is not without limitations. First, we were only able to ensure equal distribution to the two experimental conditions (congruent vs. incongruent), but not to the stimulus material ultimately presented (in favor vs. against), as this also depended on participants’ prior opinions. Since, for both topics, more participants indicated supportive than opposing opinions, more participants in the congruent condition received the positive stimulus material and more participants in the incongruent condition received the opposing stimulus material. Although we carefully designed the two stimulus sets to be as similar as possible (by ensuring the same word count as well as analogous sentence structure and wording of the comments in both sets), we still cannot rule out confounding effects of stimulus valence. Furthermore, due to imbalanced dropout rates in the attention check, it could be that the congruent condition attained higher data quality than the incongruent condition. To ensure that no systematic *a-priori* differences between the two conditions caused our results, we re-ran all analyses with prior opinion as a covariate which resulted in the same pattern of evidence (see Supplementary Material). In our opinion, this secures the conclusions drawn in this paper.

Second, only the first part of our study was designed experimentally. The hypotheses regarding the relationship between subjective knowledge and political participation, as well as processing fluency/perceived controversiality and subjective knowledge, can only be interpreted as correlational evidence. Therefore, we cannot test for a mediation sequence, even though our theoretical model implies this sort of causality. This underlines the need for more experimental studies that explicitly test these causal effects. We also did not include a control condition and therefore cannot judge whether the group differences are caused by an increase after receiving congruent or a decrease after receiving incongruent information, although our within-subject analyses do provide tentative evidence to this question.

Finally, the use of self-assessment scales and the concentration on social media users restricts the generalizability of our results. Future research is needed to assess whether our results replicate when more subtle or objective measures are used.

## 6. Conclusion

In summary, the present experiment provides intriguing new insights into the metacognitive effects of attitudinal (in)congruence. Attitudinally congruent information felt easier than incongruent information and resulted in a higher rating of subjective issue knowledge as well as willingness for political participation. Furthermore, subjective knowledge was related to political participation, although the causal direction of this relationship cannot be assessed by our research design. The present experiment has important practical implications. Regarding political participation, those citizens who are most politically active might not be the ones who possess the most political knowledge, but those who *feel* the best informed (Yamamoto et al., 2018; Lee et al., 2021a). This feeling of knowledge, in turn, is favored by homogeneous opinion spaces. This leads to a situation in which people who are surrounded by heterogeneous opinions, and thereby know more than their own take on an issue, feel less knowledgeable and are less willing to participate than people who solely receive reinforcing information. Inflated subjective knowledge could further discourage the search for new information and consideration of competing evidence (Rollwage et al., 2020). In the long run, this could lead to a vicious cycle fostering polarization and misinformation in homogeneous opinion spaces.

## Data availability statement

The datasets presented in this study can be found in online repositories. The names of the repository/repositories and accession number(s) can be found at: Open Science Framework, https://osf.io/52kbs/.

## Ethics statement

The studies involving human participants were reviewed and approved by Institutional ethics review board of the Department of Computer Science and Applied Cognitive Science, University of Duisburg-Essen. The patients/participants provided their written informed consent to participate in this study.

## Author contributions

LF and GN contributed to the conception and the design of the study. LF performed the statistical analyses and wrote the first draft of the manuscript. All authors contributed to the article and approved the submitted version.

## Funding

This research was supported by the Digital Society research program funded by the Ministry of Culture and Science of the German State of North Rhine-Westphalia (Grant number: 005–1709-0004; received by GN), Junior Research Group “Digital Citizenship in Network Technologies” (Project number: 1706dgn009).

## Conflict of interest

The authors declare that the research was conducted in the absence of any commercial or financial relationships that could be construed as a potential conflict of interest.

## Publisher’s note

All claims expressed in this article are solely those of the authors and do not necessarily represent those of their affiliated organizations, or those of the publisher, the editors and the reviewers. Any product that may be evaluated in this article, or claim that may be made by its manufacturer, is not guaranteed or endorsed by the publisher.

## Footnotes

^1^Please note that the wording of our dependent variable “intention to politically participate” used in hypotheses 1 and 3 deviates from our preregistered hypotheses in which we referred to “willingness for political participation.” We made this change since we believe that “intention to politically participate” better captures the construct which we measured.

^2^ https://osf.io/52kbs/
