# Supplementary material for: Metacognitive effects of attitudinal (in)congruence on social media: relating processing fluency, subjective knowledge, and political participation
Source: Front Psychol. 2023 Jul 17;14:1146674. doi: 10.3389/fpsyg.2023.1146674 (PMC10390028; doi:10.3389/fpsyg.2023.1146674)
Supplement: Supplementary file 1 [file Data_Sheet_1.docx]

Supplementary Material

Metacognitive effects of attitudinal (in)congruence on social media: Relating processing fluency, subjective knowledge, and political participation

Luna T. Frauhammer^1*^, German Neubaum^1^

*** Correspondence:** Luna T. Frauhammer, [luna.frauhammer@uni-due.de](mailto:luna.frauhammer@uni-due.de)

| Variable | *M* | *SD* | 1 | 2 | 3 |
| --- | --- | --- | --- | --- | --- |
|  |  |  |  |  |  |
| 1. Political Participation | 2.64 | 1.61 |  |  |  |
|  |  |  |  |  |  |
| 2. Subjective Knowledge | 4.56 | 1.44 | .43** |  |  |
|  |  |  | [.38, .47] |  |  |
|  |  |  |  |  |  |
| 3. Processing Fluency | 5.97 | 1.12 | -.04 | .10** |  |
|  |  |  | [-.09, .02] | [.05, .16] |  |
|  |  |  |  |  |  |
| 4. Perceived Controversiality | 4.92 | 0.92 | -.01 | .08** | .08** |
|  |  |  | [-.06, .05] | [.03, .14] | [.03, .13] |
|  |  |  |  |  |  |

**Supplementary Table 1***. Means, standard deviations, and zero-order correlations of all dependent variables*

*Note.* All values are based on the main topic data. Values in square brackets show 95% confidence intervals. * indicates *p* < .05. ** indicates *p* < .01.


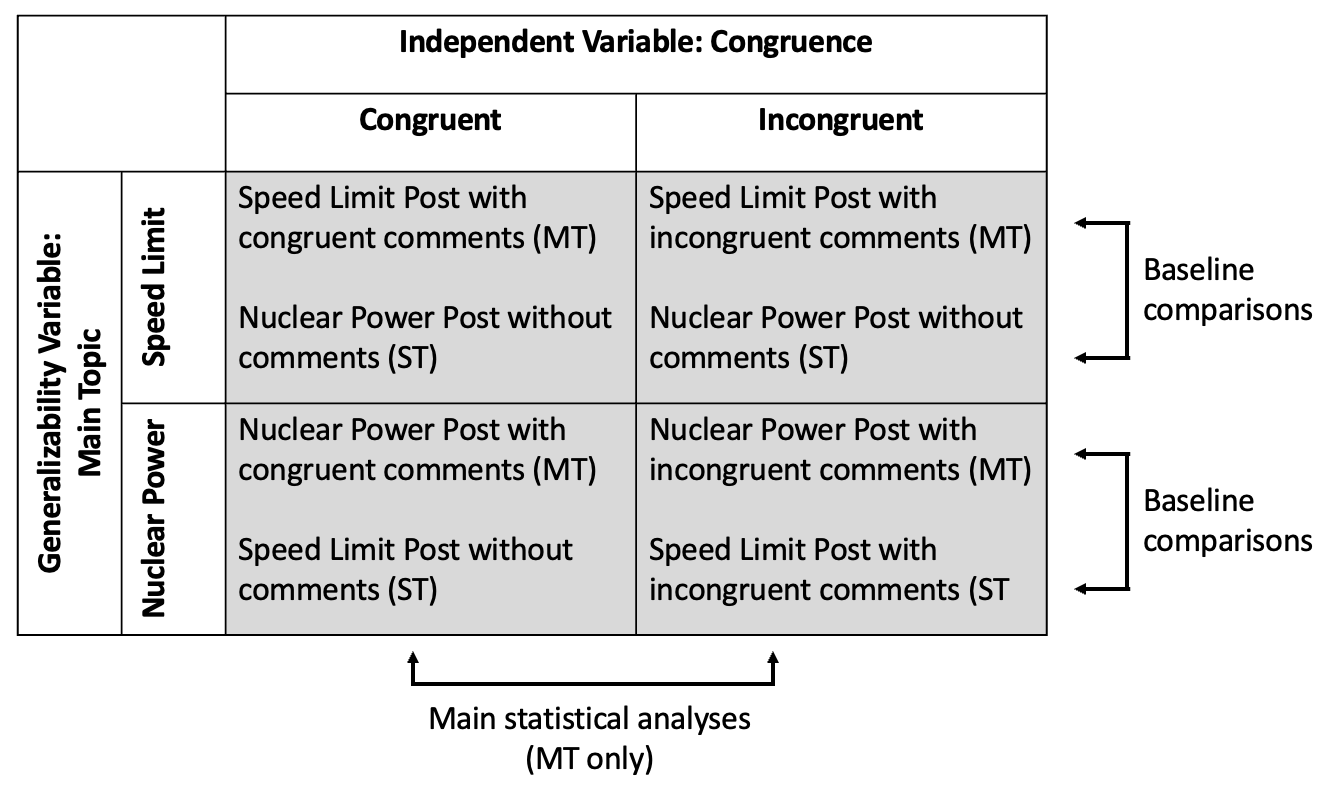


**Supplementary Figure 1.** Overview of the experimental conditions. The shaded cells show which stimulus material was presented in the four experimental groups. The arrows indicate which conditions were contrasted in the respective group comparisons. MT = main topic, ST = secondary topic.

**Attention Check Failures**

Since online panels can be prone to poor data quality (see e.g., Webb and Tangney, 2022), we included two attention checks in our experiment. The first attention check was embedded directly after stimulus presentation and asked whether the presented social media comments were rather in favor or against the discussed proposal (service life extension of nuclear power plants or implementation of a speed limit on highways, respectively). The second attention check was embedded in the political participation items and asked the participants to choose a specific answering option. Participants who failed an attention check were immediately excluded from the remaining experiment. Dropout rates were high: In total, *n* = 1,020 participants were excluded due to attention check failures (569 due to the first attention check, 451 due to the second attention check). While these high dropout rates themselves do not pose a problem per se, dropout rates in the first attention check differed systematically between the congruent and incongruent condition: *n* = 471 participants were excluded from the incongruent condition while only *n* = 98 participants were excluded from the congruent condition. Unfortunately, we can only speculate about reasons for this systematic effect, but we have two explanations that could have added up to produce the high difference. First, the participants might have only paid attention to the answering options (“rather in favor”, “rather against”) and thought that they were again being asked about their personal opinion on the issue. This would have resulted in participants in the congruent condition always passing the attention check (since the congruent condition meant that they saw user comments that conformed to their own opinion) and the participants in the incongruent condition always failing the attention check (since the incongruent condition meant that they saw user comments that contradicted their own opinion). Second, the order of the two answering options was always the same with “rather in favor” being the first and “rather against” the second option. It might be that participants who were guessing about the right answer for the attention check were more likely to choose the first answering option. Since, in total, more participants were supportive of both issues, choosing the first answering option would result in more correct answers in the congruent than in the incongruent condition.

We next evaluated whether excluded and included participants differed in their prior opinions on the issue as well as their indicated issue importance. Included and excluded participants did not differ significantly regarding their prior opinions on the respective issue, *t*(2222.6) = -0.32, *p* = 0.747. They did, however, differ significantly regarding their perceived issue relevance, *t(*2217.3) = 3.39, *p* < .001. This further led to a marginally significant difference in perceived issue relevance between the two conditions (congruent vs. incongruent), *t*(1255.7) = 1.95, *p* = .051, *d* = 0.11.

To ensure that our results were not corrupted by systematic differences between the two conditions which might have been caused by the differing dropout rates, we reran our main analyses of the hypotheses 1, 2, and 4 while controlling for the participants’ prior opinions and perceived issue relevance. All originally found results remained significant. **Political participation (H1):** *b*_Condition_ = -0.30, *t*(1254) = -3.51, *p* < .001, *b*_PriorOpinion_ = 0.02, *t*(1254) = 1.04, *p* = .297 , *b*_Relevance_ = 0.28, *t*(1254) = 11.48, *p* < .001. **Subjective knowledge (H2):** *b*_Condition_ = -0.26, *t*(1254) = -3.46, *p* < .001, *b*_PriorOpinion_ = 0.03, *t*(1254) = 1.74, *p* = .081, *b*_Relevance_ = 0.25, *t*(1254) = 11.30, *p* < .001. **Processing Fluency (H4):** *b*_Condition_ = -0.22, *t*(1254) = -3.52, *p* < .001, *b*_PriorOpinion_ = 0.02, *t*(1254) = 1.52, *p* = 0.13, *b*_Relevance_ = 0.01, *t*(1254) = 0.58, *p* = 0.563.

**Literature**

Webb, M.A., Tangney, J.P., 2022. Too Good to Be True: Bots and Bad Data From Mechanical Turk. Perspect. Psychol. Sci. 174569162211200. https://doi.org/10.1177/17456916221120027
